# Climate Zone of Geographical Origin Associations with Essential Oil Composition, Yield, and Chemotype Distribution in *Coriandrum sativum* L.: A Multivariate Analysis of 48 Global Accessions

**DOI:** 10.3390/molecules31111950

**Published:** 2026-06-04

**Authors:** Minju Kim, Songmun Kim

**Affiliations:** 1 Convergence Program of Cosmetic Science, Kangwon National University, Chuncheon 24341, Gangwon-do, Republic of Korea; 2 School of Natural Resources and Environmental Science, Kangwon National University, Chuncheon 24341, Gangwon-do, Republic of Korea

**Keywords:** *Coriandrum sativum*, essential oil, chemotype, PCA, PERMANOVA, germplasm, climate zone, linalool, (E)-2-decenal, geographical origin

## Abstract

*Coriandrum sativum* L. is a widely cultivated aromatic herb exhibiting substantial variation in essential oil quality and yield among different accessions. This study assessed germination performance, essential oil composition, yield, chemotype distribution, and fragrance characteristics in 48 *C. sativum* accessions collected from 19 countries spanning four Köppen–Geiger climate zones: Tropical/Subtropical, Arid/Semi-arid, Temperate, and Continental/Cold. All accessions were grown under standardized field conditions, and essential oils were extracted from aerial parts using steam distillation followed by direct-GC/MS analysis. Seed germination rates were consistently high (mean: 92.25 ± 5.85%; range: 71–100%) and did not differ significantly by climate zone (Kruskal–Wallis H = 5.500, *p* = 0.139) or country of origin (H = 21.833, *p* = 0.240), indicating that post-harvest management, rather than climatic provenance, primarily determines seed viability. Essential oil profiles were dominated by (E)-2-decenal (mean: 44.56%), decanal (11.75%), and 2-dodecenal (13.47%). Principal component analysis (PCA) of 18 compounds detected in at least 19 accessions accounted for 70.16% of total variance across five components, with PC1 reflecting a gradient from long-chain saturated aldehyde accumulation to linalool enrichment. Permutational multivariate analysis of variance (PERMANOVA) demonstrated significant compositional differentiation among climate zones (Pseudo-F = 1.662, *p* = 0.028), whereas country-level grouping was not significant (*p* = 0.256). Tropical/subtropical accessions exhibited the highest linalool content (mean: 15.39 ± 8.71%) and essential oil yield (mean: 0.269 ± 0.120% *v*/*w*), significantly surpassing arid/semi-arid and temperate zones (*p* < 0.05). Two chemotypes were identified, (E)-2-decenal (91.7%) and linalool (8.3%), each associated with distinct fragrance profiles (earthy/aldehydic/woody versus herbal/sweet, respectively). These findings demonstrate that climate zone of origin is significantly associated with *C. sativum* essential oil composition and productivity, with tropical/subtropical accessions providing superior yield and linalool content. Chemotype characterization offers an additional criterion for germplasm selection in targeted industrial applications.

## 1. Introduction

*Coriandrum sativum* L. (coriander; Apiaceae) is one of the oldest cultivated aromatic herbs, with documented use spanning millennia throughout Asia, the Mediterranean, and the Middle East. In English, “cilantro” refers to the fresh leaves, while “coriander” denotes the dried fruits and seeds. The species plays important roles in culinary and traditional medicinal practices across various cultures, including Ayurvedic medicine in South Asia and herbal pharmacopoeias in East and Southeast Asia. Its global importance as both a spice and medicinal resource has led to extensive scientific investigation of the chemical composition and biological activities of its essential oil. *C. sativum* essential oil has been reported to exhibit antimicrobial, antioxidant, and anti-inflammatory activities, largely attributable to its major volatile constituents [1,2].

*C. sativum* essential oil has been extensively studied for its antimicrobial, antifungal, antioxidant, and insecticidal properties, with biological activity closely linked to volatile composition [1,3,4]. Considerable variation in essential oil composition has been reported across cultivars, developmental stages, and geographical origins, indicating the necessity of systematic evaluation under controlled conditions [1,3,5]. In particular, chemotypic diversity—defined by the dominance of either linalool or aliphatic C10–C12 aldehydes—has been recognized as a key determinant of end-use suitability, yet its relationship to broad climatic provenance across globally diverse germplasm remains poorly understood [1,3,4,5].

The composition of the essential oil, however, differs markedly depending on the plant organ from which it is extracted: fruit (seed)-derived oils are characteristically dominated by the monoterpene alcohol linalool, whereas oils extracted from aerial parts (leaves and stems) are rich in aliphatic C10–C12 aldehydes, particularly (E)-2-decenal and decanal [4,6]. This organ-specific compositional divergence has important implications for industrial applications, as the two oil types confer distinct aromatic profiles suited to different end uses in the food, fragrance, and pharmaceutical sectors.

Beyond organ-specific variation, the chemical composition and essential oil yield of *C. sativum* display significant diversity among accessions from different geographical origins. Saxena et al. documented substantial variation in linalool content (16.59–96.69%) among 148 Indian germplasm accessions, while Nejad Ebrahimi et al. reported wide inter-accession variation (40.9–79.9%) within Iranian collections, despite similar climatic backgrounds [5,7]. These observations suggest that both genetic and environmental factors contribute to compositional diversity. However, the relative impact of broad climatic factors, compared to country- or accession-level variables, has not been systematically assessed across globally diverse germplasm. Furthermore, most previous studies have examined essential oil composition or agronomic traits such as yield and germination in isolation, leaving the integrated relationships among provenance, seed viability, oil productivity, chemotype identity, and fragrance characteristics insufficiently explored.

The sensory character of *C. sativum* is also shaped by its volatile chemistry. The characteristic “cilantro” aroma of aldehyde-rich accessions and the floral-herbal quality of linalool-rich types have distinct industrial relevance, and individual variation in olfactory receptor gene expression (OR6A2) is known to modulate consumer perception of these aromas [8], further motivating the need for precise chemical characterization of diverse germplasm collections.

This study aimed to comprehensively characterize 48 *C. sativum* accessions from 19 countries representing four Köppen–Geiger climate zones. The objectives were: (i) to assess seed germination viability prior to cultivation and its relationship with geographical origin; (ii) to characterize essential oil composition of aerial-part extracts using direct-GC/MS and identify principal axes of compositional variation via principal component analysis (PCA); (iii) to determine whether climate zone of origin significantly structures essential oil composition using permutational multivariate analysis of variance (PERMANOVA); (iv) to quantify essential oil yield and evaluate its dependence on climatic provenance; and (v) to classify accessions by chemotype and describe associated fragrance profiles, thereby informing germplasm selection for targeted industrial applications.

## 2. Results

### 2.1. Germination Performance of C. sativum Accessions

Germination performance of 48 *C. sativum* accessions sourced from 19 countries was assessed prior to field establishment (Table 1). Overall, seeds exhibited consistently high viability, with a mean germination rate of 92.25 ± 5.85% (median: 93.0%; IQR: 89.0–96.2%), and individual accession rates ranging from 71% to 100%. The low coefficient of variation (CV = 6.35%) across all accessions reflects a collectively high and homogenous level of seed viability, indicating that the germplasm collection was suitable for downstream agronomic and phytochemical analyses.

At the accession level, seeds from Peru (100%), Bhutan (99%), and Kyrgyzstan (98%) achieved the highest recorded germination rates, while Russian-origin accessions exhibited the widest inter-accession variability (71–91%; mean: 81.0 ± 14.1%), with one accession (CS06) recording the lowest germination rate observed in this study (71%). Among countries represented by multiple accessions, Uzbekistan (*n* = 7) demonstrated notably consistent germination performance (mean: 94.6 ± 3.2%; range: 90–100%), whereas Korea (*n* = 9) showed greater intra-country variation (mean: 91.3 ± 7.0%; range: 76–99%).

### 2.2. Germination Rates Across Climate Zones

To assess whether broad climatic conditions at the seed origin influenced germination capacity, accessions were classified into four Köppen–Geiger climate zones: tropical/subtropical, arid/semi-arid, temperate, and continental/cold (Table 2). Accessions from Arid/Semi-arid regions (*n* = 13; mean: 94.23 ± 3.88%) and tropical/subtropical zones (*n* = 11; mean: 93.73 ± 5.69%) exhibited the highest median germination rates (both 94.0%), while Temperate-zone accessions (*n* = 22; mean: 91.09 ± 5.18%) showed marginally lower performance. Continental/Cold accessions (*n* = 2; Russia only) recorded the lowest mean germination rate (81.00 ± 14.14%), though the extremely limited sample size precludes generalization.

The Kruskal–Wallis test indicated no significant differences in germination rates among climate zones (H = 5.500, *p* = 0.139), consistent with the country-level analysis (H = 21.833, *p* = 0.240). Spearman correlation also showed no association between latitude of origin and germination rate (ρ = −0.125, *p* = 0.396). The consistently high germination performance across diverse geographical origins (overall mean: 92.25 ± 5.85%) suggests that seed viability in C. sativum is not primarily determined by climatic provenance, but rather reflects the standardized post-harvest management applied to all accessions. Therefore, all 48 accessions were included in subsequent analyses of essential oil composition.

### 2.3. Essential Oil Composition and Principal Component Analysis

#### 2.3.1. Overview of Volatile Composition

The essential oil composition of 48 *C. sativum* accessions was characterized by direct-GC/MS, and a total of 20 volatile compounds were detected across the 48 accessions (Supplementary Table S1); of these, **18** compounds present in 19 or more accessions were selected for multivariate analysis (Table 3). Among these, (E)-2-decenal (C2) was the most abundant compound across all accessions (overall mean: 44.56%), followed by 2-dodecenal (C5; mean: 13.47%) and decanal (C1; mean: 11.75%), consistent with the characteristic aldehyde-rich profile reported for *C. sativum* essential oil. Seven compounds (C8, C9, C12, C14–C17) with |ΔRI| > 20 but MS match factor ≥ 80% and detection in ≥19 accessions were retained as tentatively identified (^†^; Supplementary Table S2). Linalool (C7), a monoterpene alcohol, exhibited the highest inter-accession variability, ranging from 0% to 61.12% across the collection, and was identified as a key driver of compositional differentiation among accessions (Supplementary Table S1).

#### 2.3.2. Principal Component Analysis (PCA)

**Figure 1:**
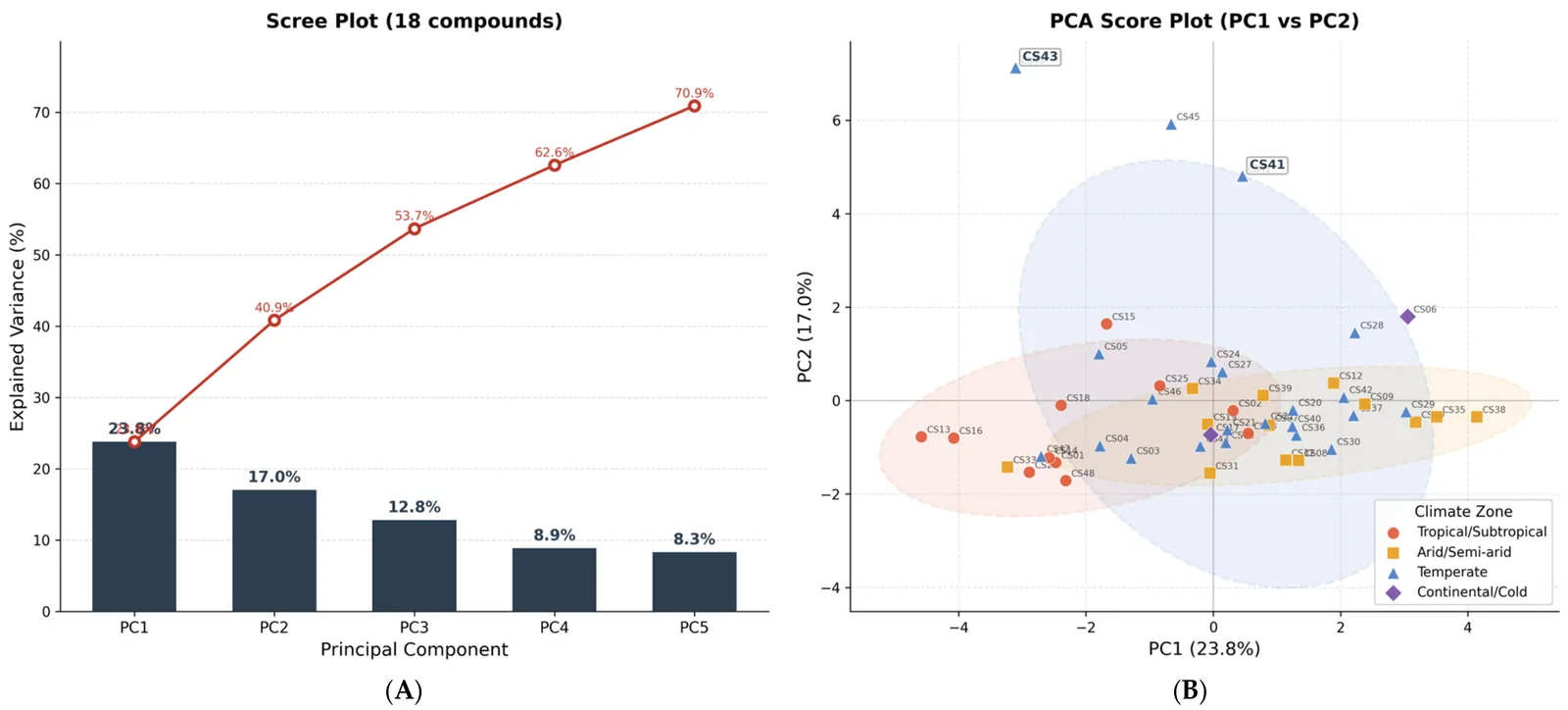
(**A**) Scree plot showing variance explained by each principal component. (**B**) PCA score plot of 48 *C. sativum* accessions based on z-score standardized relative content of 18 volatile compounds. Points are colored by Köppen–Geiger climate zone. Ellipses represent 95% confidence intervals for each climate zone group (bivariate normal assumption). CS41 and CS43 (Linalool chemotype) are individually labeled.

PCA was performed on standardized (z-score) content data for the 18 compounds detected in ≥19 accessions (48 accessions × 18 variables). The first five principal components (PC1–PC5) collectively explained 70.16% of the total variance, indicating broad compositional diversity within the accession collection (Table 4; Figure 1).

The relatively low combined variance of PC1 and PC2 (41.1%) is characteristic of high-dimensional volatile composition datasets, where multiple partially correlated biosynthetic pathways distribute variance across many components. The five-component solution (70.16%) was retained for interpretation, and the PC1–PC2 plot was used solely to visualize the primary axis of climate zone differentiation. Two-component variance in comparable *C. sativum* essential oil PCA studies typically ranges from 35–55% [4,5], confirming that the present result is within the expected range.

**Figure 2:**
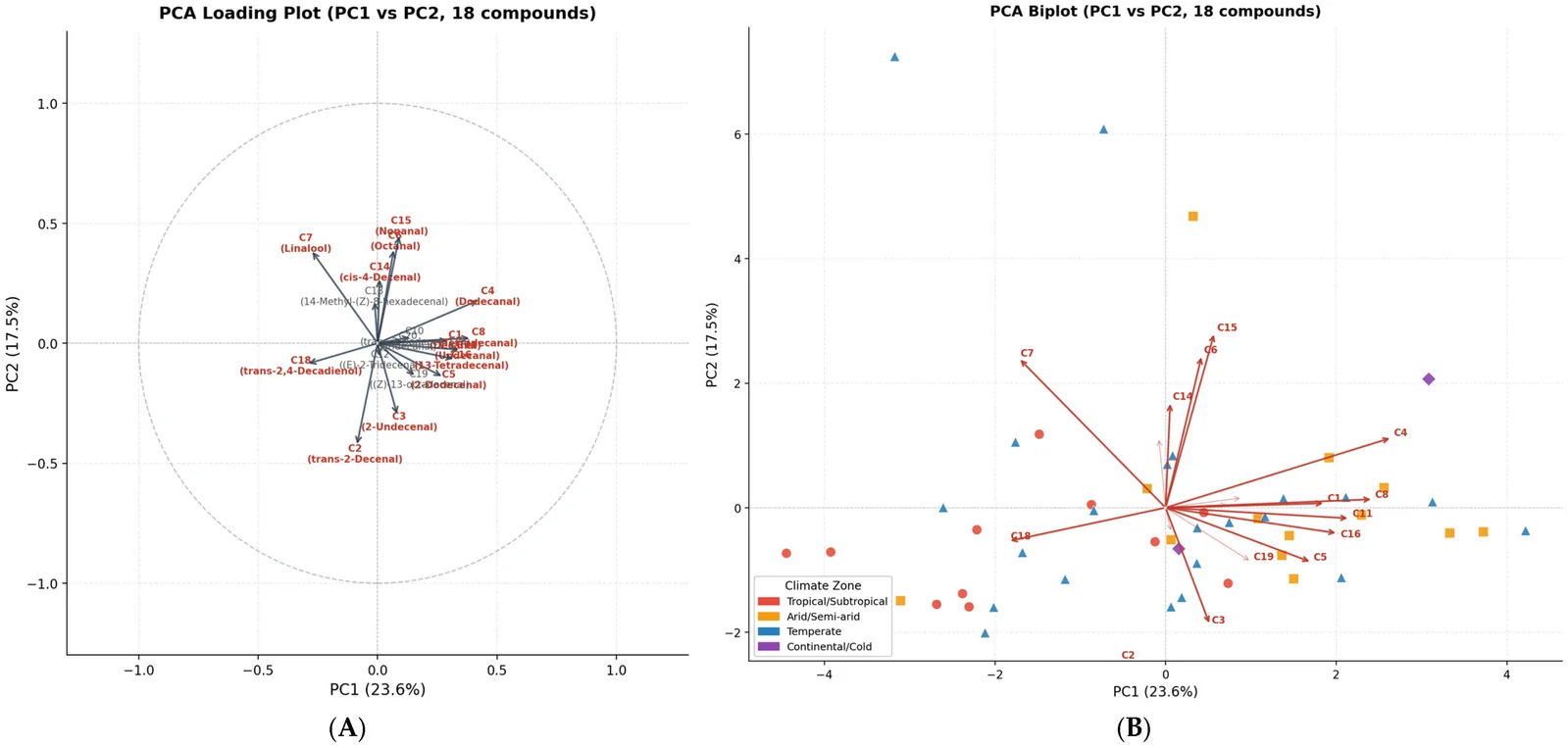
(**A**) PCA loading plot of 18 volatile compounds on PC1 and PC2 (C1–C18 as defined in Table 5). (**B**) Biplot of accession scores and compound loadings; arrow length and direction indicate the magnitude and axis of maximum contribution.

PC1 (23.56% of variance) represented a gradient of saturated long-chain aldehyde accumulation (Figure 2A). The principal positive contributors to PC1 were dodecanal (C4; loading: +0.431), tetradecanal (C8; +0.395), undecanal (C10; +0.351), decanal (C1; +0.304), and 13-tetradecenal (C15; +0.328), all of which are saturated or monounsaturated C8–C14 aldehydes. Accessions with high positive PC1 scores showed elevated concentrations of these heavy aliphatic aldehydes. Conversely, *trans*-2,4-decadienol (C16; −0.300) and linalool (C7; −0.280) loaded negatively on PC1, indicating an inverse relationship between the accumulation of long-chain saturated aldehydes and the relative proportions of these oxygenated terpene and diene-alcohol compounds.

PC2 (17.46% of variance) was dominated by an antagonistic contrast between short-chain aldehydes/terpenes and medium-chain unsaturated aldehydes. Nonanal (C14; +0.457), linalool (C7; +0.390), and octanal (C6; +0.398) loaded positively, whereas (E)-2-decenal (C2; −0.431), 2-undecenal (C3; −0.306), and 2-dodecenal (C5; −0.193) loaded negatively. Accessions with high PC2 scores were thus enriched in volatile monoterpenes and C8–C9 aldehydes relative to the dominant C10–C12 unsaturated aldehydes.

#### 2.3.3. Geographical Differentiation of Essential Oil Composition

Visualization of PC1 vs. PC2 scores, color-coded by Köppen–Geiger climate zone of accession origin, revealed a partial but discernible spatial structure in the score plot (Figure 1B; Figure 2B for biplot overlay). Tropical/subtropical accessions (*n* = 11) were consistently displaced towards the negative end of PC1 (centroid: PC1 = −1.752, PC2 = −0.627), reflecting their comparatively lower content of long-chain saturated aldehydes and higher relative linalool concentrations (mean: 15.39 ± 8.71%) relative to arid/semi-arid (mean: 4.44 ± 9.45%; centroid: PC1 = +1.251) and continental/cold accessions (mean: 0.58 ± 0.66%; centroid: PC1 = +1.618). Temperate-zone accessions (*n* = 22) occupied an intermediate position along PC1 (centroid: PC1 = −0.010), albeit with considerable within-group dispersion attributable to several Korean accessions (CS41 and CS43) that exhibited exceptionally high linalool content (61.12% and 45.25%, respectively), driving their scores to extreme negative PC1 values.

The ecological and chemical significance of this compositional separation along PC1 is further supported by the contrasting concentrations of decanal (C1), the highest-loading PC1 compound after dodecanal. Decanal content was markedly lower in tropical/subtropical accessions (mean: 7.96 ± 3.48%) compared to arid/semi-arid (14.67 ± 5.39%) and continental/cold accessions (18.23 ± 4.06%), suggesting that accessions from warmer, more humid climates tend to biosynthesize proportionally less of the long-chain aliphatic aldehydes that dominate the essential oil of most accessions (Table 5).

To statistically confirm the climate zone-level differentiation observed in the score plot, a PERMANOVA was conducted on the full 18-compound standardized dataset using Euclidean distance with 9999 permutations (Table 6). Climate zone explained a significant proportion of compositional variance (Pseudo-F = 1.662, *p* = 0.028), indicating that essential oil profiles differed significantly among the four climate zone groups. In contrast, when accessions were grouped at the country level (19 groups), PERMANOVA yielded a non-significant result (Pseudo-F = 1.130, *p* = 0.256), suggesting that compositional differentiation is more coherently structured at the level of broad climatic regions than at the level of individual countries of origin. These findings collectively indicate that the climatic environment at the geographical origin of *C. sativum* accessions is associated with systematic differences in essential oil composition, particularly in the relative balance between long-chain aliphatic aldehydes and linalool, even though germination viability was unaffected by these same geographical factors (Section 3.1).

### 2.4. Essential Oil Yield, Chemotype Distribution, and Fragrance Characteristics

#### 2.4.1. Essential Oil Yield

The essential oil yield of 48 *C. sativum* accessions, extracted by SDE, ranged from 0.062% to 0.446% (*v*/*w*), with an overall mean of 0.158 ± 0.092% (median: 0.140%). A Kruskal–Wallis test revealed highly significant differences in yield among climate zones (H = 19.119, df = 3, *p* = 0.0003) (Table 7). Post hoc pairwise comparisons with Bonferroni correction indicated that Tropical/Subtropical-origin accessions (mean: 0.269 ± 0.120% *v*/*w*) yielded significantly more essential oil than both Arid/Semi-arid (mean: 0.111 ± 0.059%; *p* = 0.0020) and Temperate accessions (mean: 0.133 ± 0.037%; *p* = 0.0021), while no significant differences were detected among the remaining zone pairs. This pattern indicates that accessions from warm, humid climates exhibit substantially greater essential oil accumulation, consistent with the known role of elevated temperature and humidity in stimulating secondary metabolite biosynthesis in aromatic plants.

The two highest-yielding accessions were CS16 (Peru; 0.446% *v*/*w*) and CS18 (Myanmar; 0.411% *v*/*w*), both originating from the Tropical/Subtropical zone. In contrast, the lowest yields were observed in CS35 (Kazakhstan; 0.062%) and CS10 (Uzbekistan; 0.069%), both from the Arid/Semi-arid zone. This pronounced yield contrast across climatic extremes highlights the potential for geographically targeted germplasm selection in breeding programs aimed at maximizing essential oil productivity.

#### 2.4.2. Chemotype Distribution

Based on the dominant compound in each accession’s essential oil profile, two chemotypes were identified across the 48 accessions (Table 8). The (E)-2-decenal chemotype was predominant, comprising 44 accessions (91.7%), while the Linalool chemotype was identified in four accessions (8.3%): CS15 (India; Linalool = 30.44%), CS39 (Mongolia; 34.52%), CS41 (Korea; 61.12%), and CS43 (Korea; 45.25%). The Linalool chemotype was distributed across three climate zones—Tropical/Subtropical, Arid/Semi-arid, and Temperate—indicating that this chemotypic variant is not restricted to a specific climatic provenance but may arise from accession-specific genetic or environmental factors.

#### 2.4.3. Fragrance Characteristics

The fragrance profile of each accession’s essential oil was characterized across 12 descriptive fragrance types (Table 8 and Table 9). Across all 48 accessions, earthy (65%), aldehydic (60%), and woody (52%) were the three most frequently assigned fragrance descriptors, reflecting the dominance of aliphatic aldehyde compounds in the overall collection. The two chemotypes exhibited distinct fragrance signatures. The (E)-2-Decenal chemotype was predominantly characterized by earthy (64%), aldehydic (61%), and woody (57%) notes, consistent with the heavy aliphatic aldehyde-dominant composition of these accessions. In contrast, all four Linalool-chemotype accessions (100%) were assigned the herbal descriptor, and the majority also exhibited sweet (75%) and earthy (75%) notes, reflecting the characteristic floral-herbaceous quality of linalool-rich essential oils. Oriental and oily notes, by contrast, were absent from all Linalool-chemotype accessions.

Taken together, the chemotype and fragrance data provide a functionally relevant complement to the PCA and PERMANOVA results. While PERMANOVA identified climate zone as a significant structuring factor for overall composition (*p* = 0.028), chemotype classification demonstrates that the most pronounced intra-zone variation, especially within the Temperate zone, is due to a small subset of Linalool-rich accessions with distinct fragrance profiles. This distinction has direct practical implications: (E)-2-decenal-type accessions, characterized by earthy and aldehydic notes, are more suitable for culinary and savory flavoring applications, whereas Linalool-type accessions, with herbal and sweet notes, may be preferentially selected for cosmetic fragrance or nutraceutical use.

## 3. Discussion

### 3.1. Germination Viability Across Geographical Origins

The uniformly high germination rates observed across 48 *C. sativum* accessions from 19 countries (mean: 92.25 ± 5.85%) demonstrate that the germplasm collection maintained robust seed viability irrespective of geographical origin. The absence of statistically significant differences between countries (H = 21.833, *p* = 0.240) and climate zones (H = 5.500, *p* = 0.139) suggests that seed viability in *C. sativum* is not systematically determined by the climatic conditions of the accession’s origin. The extreme inter-accession variability observed within the Russian accessions (71–91%)—particularly the outlier CS06 (71%)—more plausibly reflects accession-specific post-harvest factors such as seed maturity at harvest, mechanical damage, or storage history rather than any systematic climatic effect, given that the co-sourced Russian accession CS17 achieved 91% germination.

### 3.2. Essential Oil Composition and Geographical Differentiation

The essential oil profiles of the 48 accessions were dominated by (E)-2-decenal (mean: 44.56%), decanal (11.75%), and 2-dodecenal (13.47%), which is in line with the aliphatic aldehyde-rich composition of coriander aerial-part essential oil of *C. sativum* that has been extensively documented and further synthesized in global phytochemical profiles [4,9,10,11]. The PERMANOVA results revealed that essential oil composition was significantly structured by climate zone of origin (Pseudo-F = 1.662, *p* = 0.028), whereas country-level grouping yielded a non-significant result (*p* = 0.256). This indicates that broad climatic conditions—rather than national provenance per se—show a stronger association with compositional variation, likely reflecting shared agroclimatic growing environments across countries within the same climate zone.

The primary axis of compositional variation (PC1, 23.56%) represented a gradient from long-chain saturated aldehyde accumulation (dodecanal, tetradecanal, decanal) toward linalool-dominant profiles. Tropical/Subtropical accessions were consistently displaced toward the linalool-enriched end of PC1, with a zone mean of 15.39 ± 8.71% for linalool, compared to 0.58 ± 0.66% in Continental/Cold accessions. This pattern echoes findings by Saxena et al., who evaluated essential oil composition across 148 *C. sativum* germplasm lines collected from diverse geographical locations in India and identified substantial variation in linalool content (16.59–96.69%), which similarly clustered into compositionally distinct groups based on origin [5]. Notably, their observation of a strong negative correlation between linalool and geranyl acetate content (r = −0.811) structurally parallels the inverse relationship between linalool and long-chain aliphatic aldehydes along PC1 in the present study, suggesting that the antagonistic accumulation of terpenoid alcohols versus aliphatic carbonyl compounds may represent a conserved compositional trade-off in *C. sativum* essential oil biosynthesis across diverse germplasm collections. Environmental factors are thought to influence coriander essential oil production, with temperature variations being related with alterations in volatile composition, including changes in linalool abundance [1]. The partition between terpenoid alcohols and long-chain aliphatic carbonyls is altered by changing environmental parameters, as demonstrated by the climate-driven variance and thermal sensitivity in volatile accumulation [12]. This further supports the idea that macro-environmental variations significantly influence phenotypic plasticity in *C. sativum* volatile profiles. A limitation of this study is that nine countries were represented by single accessions, introducing sampling contingency at the country level. This further supports prioritizing climate zone-level grouping, where replication was sufficient (*n* = 11–22) for meaningful statistical inference.

### 3.3. Chemotype Distribution and Intra-Zone Variation

Two chemotypes were identified across the 48 accessions: the (E)-2-decenal chemotype (91.7%) and the Linalool chemotype (8.3%). The Linalool chemotype was not confined to a single climate zone, appearing in accessions from India (Tropical/Subtropical), Mongolia (Arid/Semi-arid), and Korea (Temperate), suggesting that this chemotypic variant is determined at the accession level by genetic factors rather than by broad climatic provenance alone. This interpretation is supported by Nejad Ebrahimi et al., who reported substantial inter-accession variation in linalool content (40.9–79.9%) among *C. sativum* accessions collected from different regions within Iran—a single country spanning predominantly Arid/Semi-arid climatic conditions—demonstrating that linalool accumulation can vary widely even among accessions sharing a common climatic background [7]. Consistently, a comprehensive multi-cultivar evaluation was confirmed that while accessions from highly diverse geographical provenances uniformly maintained a primary β-linalool chemotype, their narrow-sense chemical constituents varied distinctly independent of broad national labels, pointing to strict cultivar-specific genetic controls [11]. The absence of clear geographical structuring of chemotype distribution observed in the present study is therefore consistent with the view that linalool biosynthetic capacity in *C. sativum* is governed primarily by accession-specific genetic constitution rather than by regional climate per se [2]. The two Korean Linalool-chemotype accessions (CS41: 61.12%; CS43: 45.25%) substantially increased within-group dispersion in the Temperate cluster on the PCA score plot, highlighting the confounding effect that rare high-linalool accessions can exert on region-level multivariate analyses. This observation underscores the importance of chemotype-level characterization as a complement to geographical grouping in germplasm studies.

### 3.4. Essential Oil Yield and Its Relationship to Climatic Origin

Essential oil yield varied markedly across accessions (0.062–0.446% *v*/*w*), with Tropical/Subtropical-origin accessions yielding significantly more oil than those from Arid/Semi-arid and Temperate zones (Kruskal–Wallis H = 19.119, *p* = 0.0003). That climatic conditions exert a significant influence on *C. sativum* essential oil yield is well established: Shams et al. demonstrated that environmental factors—particularly altitude and precipitation—significantly affected oil content and dry matter yield in coriander cultivated across climatically distinct sites in Iran, confirming that yield variation in this species is strongly climate-dependent [13]. The higher productivity observed in Tropical/Subtropical accessions in the present study is further consistent with the known stimulatory effect of elevated temperature and relative humidity on essential oil biosynthesis and secretory gland density in aromatic plants more broadly [14]. The substantially higher yields in accessions from Peru (CS16: 0.446%) and Myanmar (CS18: 0.411%), both from tropical climates, relative to the lowest-yielding Arid/Semi-arid accessions (Kazakhstan CS35: 0.062%; Uzbekistan CS10: 0.069%), suggest that the thermal and hydrological conditions experienced during seed formation in the country of origin may have lasting epigenetic or physiological effects on secondary metabolite capacity—a phenomenon observed in other Apiaceae species [15]. From a practical standpoint, the significantly higher essential oil productivity of Tropical/Subtropical-origin accessions, when combined with the consistently high germination performance observed across all zones, positions these accessions as particularly attractive candidates for selection in essential oil-focused breeding programs. It should be noted that the Continental/Cold zone was represented by only two accessions (both from Russia), which limits the generalizability of findings for this climatic category. To present a more thorough description of cold-climate germplasm, future research should include accessions from Siberia, Northern Europe, or high-latitude areas of North America.

### 3.5. Fragrance Characteristics and Practical Implications

The chemotype-dependent divergence in fragrance profiles—earthy/aldehydic/woody for (E)-2-Decenal-type versus herbal/sweet for Linalool-type accessions—directly reflects the contrasting chemical composition of these groups and has practical implications for end-use selection. The prevalence of heavy aliphatic aldehyde notes (earthy, aldehydic) in (E)-2-Decenal-type accessions corresponds with consumer preference data for culinary coriander in European and Asian markets, where the characteristic “cilantro-like” aroma from C6–C12 aldehydes is a primary quality criterion [16]. In contrast, the herbal and sweet character of Linalool-type accessions aligns with fragrance profiles valued in cosmetic and nutraceutical applications, where linalool is a key ingredient for its calming and antimicrobial properties [17]. Furthermore, accurate screening of these minor and significant substance changes is essential for improving *C. sativum* raw materials in international perfumery and pharmaceutical formulations, as demonstrated by recent validation of important commercial chemotypes [1,9]. These results indicate that the 48-accession collection possesses phenotypic diversity suitable for differentiated industrial applications, and that chemotype screening, rather than geographic selection alone, should be incorporated into *C. sativum* germplasm utilization strategies.

## 4. Materials and Methods

### 4.1. Collection and Cultivation of Plant Samples

Seeds from 48 *C. sativum* accessions were obtained from the National Agrobiodiversity Centre (Chuncheon, Republic of Korea). All accessions were cultivated simultaneously at a single location—GARES experimental field, Chuncheon, Gangwon-do, Korea (N 37°55′45.4″; E 127°43′44.2″)—during the same growing season. Seedlings were maintained for 35–36 days in a glass greenhouse under uniform controlled conditions (23–25 °C, 50% relative humidity). Essential oils were extracted at the same growth stage across all accessions. All samples were cultivated at the same location in Chuncheon, Gangwon-do, Korea (N 37°55′45.4″; E 127°43′44.2″). Not all plant samples were included in the study; essential oils could not be extracted from some plants due to insufficient growth or other issues, resulting in a final dataset of 48 accessions for analysis. Nine of the 19 countries were represented by a single accession (Bulgaria, China, Bhutan, Costa Rica, India, Kazakhstan, Kyrgyzstan, Ukraine, and United States), precluding assessment of intra-country variability. These accessions were therefore included in climate zone-level analyses only and were not used for country-level comparisons (Table 10).

### 4.2. Essential Oil Extraction

Using a steam distillation extraction (SDE) method, essential oils from the aerial parts of 48 *C. sativum* populations were isolated individually. An apparatus for steam distillation (EssenLab Plus, Hanil Lab Tech Co., Ltd., Yangju, Korea) was used to steam distill the plant samples for 90 min at boiling temperature. The weight of each fresh sample was used to calculate the essential oil yield (%, *v*/*w*). The collected essential oil was dried over anhydrous sodium sulfate to remove residual water prior to storage. Pure essential oil was stored at 4 °C prior to GC-MS analysis. The fragrance profile of each essential oil was assessed by three trained evaluators independently, each with prior experience in sensory evaluation of aromatic plant materials. Descriptors were assigned by consensus, and each evaluator scored independently using 12 standardized fragrance descriptors: aldehydic, citrus, earthy, floral, herbal, medicinal, oily, oriental, peppery, spicy, sweet, and woody. Color was recorded visually upon extraction.

### 4.3. Gas Chromatography-Mass Spectrometry (GC-MS) Analysis

Varian (Palo Alto, CA, USA) supplied a Varian CP3800 gas chromatograph and a Varian 1200L MS detector for the identification and quantitative analysis of the constituents of the essential oils. A VF-5MS (Agilent, Santa Clara, CA, USA) polydimethylsiloxane (30 m × 0.25 mm × 0.25 μm) capillary column was used for the GC-MS analysis. Helium was used as the carrier gas at a flow rate of 1 mL/min. The GC conditions were as follows: the oven was set to operate between 50 °C and 250 °C, increasing at 5 °C/min. The initial hold duration was set to 5 min, and the final hold time to 1 min. The injection volume was 1 μL with a split ratio of 10:1. The MS conditions were as follows: the ion source temperature was set to 200 °C, electron beam energy was set to 70 eV, ionization mode was electron ionization, and mass scan range was set to 50–500 *m*/*z*. Compounds were identified by matching experimental retention indices (RI), calculated relative to a homologous series of n-alkanes (C8–C20) on a VF-5MS nonpolar column, against reference values from Adams (2017) and the NIST Mass Spectral Library (version 3.0) [18]. Identification was accepted when RI deviation (ΔRI) was ≤20 units relative to Adams (2017) [18] AI values and MS library match factor was ≥80%. Compounds exceeding the ΔRI threshold but meeting the MS match criterion and detected in ≥19 accessions were retained for multivariate analysis and designated as tentatively identified (†; see Supplementary Table S2). Each accession was assigned a chemotype based on the compound with the highest relative peak area (%) in its essential oil profile.

### 4.4. Statistical Analysis

The essential oil components from the *C. sativum* accessions were subjected to principal component analysis (PCA) to interpret GC-MS data. The GC-MS data of 48 *C. sativum* essential oil samples were combined into a single dataset for this purpose. Based on their retention indices (RI), the chemical components in the dataset were sorted in ascending order. Among these, only components detected in 19 or more of the 48 accessions were selected for PCA, yielding a final dataset of **18** compounds for PCA (compounds detected in fewer than 19 accessions after final screening were excluded). Each accession was assigned a chemotype based on the compound with the highest relative peak area (%) in its essential oil profile.

For geographical comparison, accessions were classified into four climate zones according to the Köppen–Geiger climate classification system, tropical/subtropical (*n* = 11), arid/semi-arid (*n* = 13), temperate (*n* = 22), and continental/cold (*n* = 2), based on the predominant climate type of each country of origin. Differences in germination rate and essential oil yield among climate zone groups were assessed using the Kruskal–Wallis test, followed by pairwise Mann–Whitney U tests with Bonferroni correction. The relationship between the geographical latitude of the accession’s origin and germination rate was evaluated using Spearman’s rank correlation, and the effect size was estimated using eta-squared (η^2^). To test whether essential oil composition differed significantly among climate zones and countries of origin, permutational multivariate analysis of variance (PERMANOVA) was performed on the standardized compound content matrix using Euclidean distance with 9999 permutations.

All statistical analyses were performed using Python (version 3.12) with the SciPy (version 1.x) and scikit-learn (version 1.x) libraries. PCA was conducted using the “sklearn.decomposition.PCA” module on z-score standardized data. Kruskal–Wallis, Mann–Whitney U, and Spearman correlation analyses were performed using “scipy.stats.” 95% confidence ellipses for each climate zone group were computed from the mean and covariance of PC1 and PC2 scores, assuming bivariate normal distribution, and plotted using Python (matplotlib).

## 5. Conclusions

This study characterized the essential oil composition, yield, chemotype distribution, and fragrance profile of 48 *Coriandrum sativum* L. accessions from 19 countries across diverse climatic regions, as well as their pre-cultivation germination performance. Seed viability was consistently high (mean: 92.25 ± 5.85%) and not significantly associated with geographical or climatic origin, indicating that germination capacity in *C. sativum* is largely independent of provenance under standardized post-harvest conditions. In contrast, essential oil characteristics were substantially influenced by the climatic background of accession origin. PERMANOVA revealed significant compositional differentiation among Köppen–Geiger climate zones (Pseudo-F = 1.662, *p* = 0.028), with Tropical/Subtropical accessions exhibiting elevated linalool content and significantly higher essential oil yields (mean: 0.269% *v*/*w*) compared to Arid/Semi-arid and Temperate-zone accessions. The primary axis of compositional variation (PC1) represented an antagonistic gradient between long-chain aliphatic aldehyde accumulation and linalool enrichment, consistent with differential thermal regulation of the MEP pathway and fatty acid oxidation across climatic zones. Two chemotypes were identified—(E)-2-Decenal (91.7%) and Linalool (8.3%)—with the Linalool chemotype distributed across multiple climate zones, indicating accession-level genetic determination rather than climatic constraint. Chemotype identity was strongly reflected in the fragrance profile: (E)-2-Decenal-type accessions exhibited earthy, aldehydic, and woody characters suitable for culinary applications, while Linalool-type accessions displayed herbal and sweet profiles with potential for cosmetic and nutraceutical use. These findings demonstrate that climate zone of origin is meaningfully associated with *C. sativum* essential oil quality and productivity and that integrating chemotype screening with geographical provenance data can improve the efficiency of germplasm selection for targeted industrial applications.

## Figures and Tables

**Table 1:** Germination rates of *C. sativum* accessions by country of origin (100 seeds sown per accession). *n*: number of accessions per country. SD and range are not applicable (—) for single-accession countries.

Origin	Accessions (*n*)	Mean ± SD (%)	Median (%)	Range (%)
Bhutan	1	99.0	99.0	—
Bulgaria	1	84.0	84.0	—
China	1	88.0	88.0	—
Costa Rica	1	94.0	94.0	—
Georgia	1	95.0	95.0	—
India	1	97.0	97.0	—
Kazakhstan	1	96.0	96.0	—
Korea	9	91.3 ± 7.0	93.0	76–99
Kosovo	9	91.7 ± 3.2	93.0	88–97
Kyrgyzstan	1	98.0	98.0	—
Mongolia	3	93.7 ± 5.5	94.0	88–99
Myanmar	2	88.5 ± 4.9	88.5	85–92
Nepal	4	95.0 ± 4.7	94.5	91–100
Peru	1	100.0	100.0	—
Russia	2	81.0 ± 14.1	81.0	71–91
Ukraine	1	91.0	91.0	—
United States	1	87.0	87.0	—
Uzbekistan	7	94.6 ± 3.2	94.0	90–100
Vietnam	1	84.0	84.0	—

**Table 2:** Summary of germination rates of *C sativum* accessions grouped by Köppen–Geiger climate zone.

Climate Zone	*n*	Countries Included	Mean ± SD (%)	Median (%)	Range (%)
Tropical/Subtropical	11	Nepal, Bhutan, India, Myanmar, Vietnam, Costa Rica, Peru	93.73 ± 5.69	94.0	84–100
Arid/Semi-arid	13	Uzbekistan, Kazakhstan, Kyrgyzstan, Mongolia, China	94.23 ± 3.88	94.0	88–100
Temperate	22	Kosovo, Bulgaria, Georgia, Ukraine, Korea, United States	91.09 ± 5.18	92.0	76–99
Continental/Cold	2	Russia	81.00 ± 14.14	81.0	71–91

**Table 3:** Summary statistics of major volatile compounds (mean relative content, %) in Coriandrum sativum essential oils across 48 accessions.

No.	Compound	Mean (%)	SD	Min (%)	Max (%)	Detection Frequency (*n*/48)
C1	Decanal	11.75	4.77	3.73	23.59	48
C2	(E)-2-decenal	44.56	12.01	5.77	61.50	48
C3	2-Undecenal	3.30	1.09	0.76	5.57	48
C4	Dodecanal	1.46	0.48	0.32	2.67	48
C5	2-Dodecenal	13.47	4.41	3.66	25.59	48
C6	Octanal	0.57	0.28	0.03	1.51	48
C7	Linalool	8.76	13.85	0.00	61.12	46
C8–C20	Minor compounds	-	-	-	-	>19

Full compound-by-accession data are provided in Supplementary Table S1.

**Table 4:** Eigenvector loadings of 18 volatile compounds on PC1 through PC5 derived from PCA of essential oil composition of *C. sativum* accessions (*n* = 48).

No.	Compound	PC1 (23.56%)	PC2 (17.46%)	PC3 (12.92%)	PC4 (9.09%)	PC5 (7.13%)
**C1**	Decanal	+0.3040 *	+0.0115	−0.3874 *	+0.0186	−0.1874
**C2**	(E)-2-Decenal	−0.0878	−0.4308 *	−0.3139 *	+0.0431	−0.0469
**C3**	2-Undecenal	+0.0845	−0.3060 *	+0.3545 *	+0.0525	−0.2052
**C4**	Dodecanal	+0.4306 *	+0.1826	+0.0953	−0.0462	−0.0186
**C5**	2-Dodecenal	+0.2780	−0.1421	+0.4474 *	−0.0847	+0.1130
**C6**	Octanal	+0.0691	+0.3981 *	+0.0038	−0.0755	−0.1826
**C7**	Linalool	−0.2796	+0.3900 *	+0.1736	−0.0103	+0.0349
**C8**	Tetradecanal	+0.3947 *	+0.0220	+0.1605	−0.0791	+0.0996
**C9**	(2E)-Dodecen-1-ol	+0.1461	+0.0258	−0.1905	+0.5642 *	+0.1134
**C10**	Undecanal	+0.3507 *	−0.0278	−0.1218	+0.0500	−0.4841 *
**C11**	(2E)-Tridecenal	+0.0110	−0.0651	+0.4017 *	+0.4978 *	+0.1056
**C12**	14-Methyl-(Z)-8-hexadecenal	−0.0130	+0.1825	+0.1942	−0.3952 *	−0.2379
**C13**	(4Z)-Decenal	+0.0088	+0.2768	−0.1398	+0.2224	+0.2687
**C14**	Nonanal	+0.0931	+0.4568 *	−0.0407	+0.1629	−0.1411
**C15**	13-Tetradecenal	+0.3278 *	−0.0666	−0.2497	−0.2342	+0.2265
**C16**	(E,E)-2,4-Decadien-1-ol	−0.2996	−0.0873	−0.0564	−0.1642	−0.1950
**C17**	(Z)-13-Octadecenal	+0.1626	−0.1405	+0.1467	+0.0393	−0.0908
**C18**	Tridecanal	+0.1197	+0.0080	−0.0461	−0.2992	+0.5986 *

* Major contributing compounds with |loading| ≥ 0.30 (highlighted in bold blue). Explained variance: PC1 = 23.56%, PC2 = 17.46%, PC3 = 12.92%, PC4 = 9.09%, PC5 = 7.13%; cumulative PC1–PC5 = 70.16%.

**Table 5:** Mean ± SD of relative content (%) of 18 volatile compounds in *C. sativum* essential oil grouped by Köppen–Geiger climate zone of accession origin.

No.	Compound	Tropical/Subtropical (*n* = 11)	Arid/Semi-Arid (*n* = 13)	Temperate (*n* = 22)	Continental/Cold (*n* = 2)
**C1**	Decanal	7.96 ± 3.48	14.67 ± 5.39	11.34 ± 4.03	18.23 ± 4.06
**C2**	(E)-2-Decenal	45.28 ± 12.15	43.74 ± 10.70	44.80 ± 13.68	43.33 ± 9.96
**C3**	2-Undecenal	3.48 ± 1.14	3.19 ± 0.98	3.33 ± 1.15	3.13 ± 0.64
**C4**	Dodecanal	0.91 ± 0.48	1.65 ± 0.43	1.44 ± 0.48	1.74 ± 0.52
**C5**	2-Dodecenal	13.26 ± 5.87	13.57 ± 2.60	13.38 ± 4.95	14.98 ± 3.63
**C6**	Octanal	0.45 ± 0.27	0.58 ± 0.20	0.56 ± 0.29	0.85 ± 0.47
**C7**	Linalool	**15.39 ± 8.71**	4.44 ± 9.45	9.27 ± 15.52	0.58 ± 0.66
**C8**	Tetradecanal	0.25 ± 0.19	0.50 ± 0.18	0.39 ± 0.18	0.35 ± 0.49
**C** **9**	(2E)-Dodecen-1-ol	0.75 ± 0.33	1.40 ± 0.76	1.15 ± 0.69	1.08 ± 0.05
**C1** **0**	Undecanal	0.37 ± 0.32	0.91 ± 0.42	0.66 ± 0.50	0.87 ± 0.10
**C1** **1**	(2E)-Tridecenal	1.99 ± 2.93	1.57 ± 2.53	1.72 ± 2.80	2.52 ± 2.02
**C1** **2**	14-Methyl-(Z)-8-hexadecenal	0.54 ± 1.29	0.84 ± 2.45	0.68 ± 1.43	0.06 ± 0.04
**C1** **3**	(4Z)-Decenal	0.20 ± 0.25	0.26 ± 0.25	0.40 ± 0.44	0.63 ± 0.34
**C1** **4**	Nonanal	0.03 ± 0.07	0.17 ± 0.12	0.18 ± 0.19	0.23 ± 0.18
**C1** **5**	13-Tetradecenal	1.36 ± 2.50	5.69 ± 3.86	3.13 ± 3.77	5.38 ± 7.61
**C1** **6**	(E,E)-2,4-Decadien-1-ol	0.27 ± 0.31	0.16 ± 0.25	0.21 ± 0.27	0.00 ± 0.00
**C1** **7**	(Z)-13-Octadecenal	0.04 ± 0.07	0.07 ± 0.10	0.04 ± 0.07	0.09 ± 0.13
**C** **18**	Tridecanal	0.02 ± 0.04	0.05 ± 0.05	0.06 ± 0.16	0.04 ± 0.05

Values represent mean ± standard deviation. SD calculated with ddof = 0 for *n* = 2 (Continental/Cold). Highlighted in yellow (bold): highest linalool mean across climate zones.

**Table 6:** PERMANOVA results comparing essential oil composition of *C. sativum* accessions grouped by climate zone and country of origin.

Grouping Factor	Groups (k)	*n*	Pseudo-F	*p*-Value	Significance
Climate zone (Köppen–Geiger)	4	48	1.662	**0.0** **283**	*
Country of origin	19	48	1.130	0.2559	ns

* *p* < 0.05 (bold, highlighted); ns, not significant (*p* > 0.05). A total of 9999 permutations, Euclidean distance on z-score standardized data.

**Table 7:** Essential oil yield (%, *v*/*w*) of *C. sativum* accessions grouped by Köppen–Geiger climate zone. Kruskal–Wallis test: H = 19.119, df = 3, *p* = 0.0003.

Climate Zone	*n*	Mean ± SD (%, *v*/*w*)	Median	Range	Post Hoc
**Tropical/Subtropical**	11	**0.269 ± 0.120**	0.220	0.100–0.446	**a**
Arid/Semi-arid	13	0.111 ± 0.059	0.094	0.062–0.276	**b**
Temperate	22	0.133 ± 0.037	0.130	0.069–0.225	**b**
Continental/Cold	2	0.131 ± 0.035	0.131	0.106–0.156	**b**

Different letters in the Post hoc column indicate significant differences (Mann–Whitney U test with Bonferroni correction, *p* < 0.05); bold and highlight denote the zone with significantly higher essential oil yield. Continental/Cold (*n* = 2) shares letter ‘b’ with Arid/Semi-arid and Temperate but pairwise comparisons were non-significant (*p* > 0.05).

**Table 8:** Essential oil yield, color, chemotype, dominant compound area (%), and fragrance profile of 48 *C. sativum* accessions. Linalool-chemotype accessions are highlighted in yellow.

Sample	Country	Yield (%, *v*/*w*)	Color	Chemotype	Area (%)	Ald	Cit	Ear	Flo	Her	Med	Oil	Ori	Pep	Spi	Swe	Woo
**CS01**	Nepal	0.220	Orange	(E)-2-Decenal	46.45	**●**				**●**	**●**			**●**			
**CS02**	Nepal	0.170	Red	(E)-2-Decenal	45.67			**●**	**●**				**●**				
**CS03**	Kosovo	0.142	Orange	(E)-2-Decenal	57.30	**●**		**●**			**●**		**●**	**●**			
**CS04**	Korea	0.127	Milky	(E)-2-Decenal	61.50	**●**				**●**					**●**		**●**
**CS05**	Korea	0.137	Red	(E)-2-Decenal	50.82			**●**				**●**		**●**			
**CS06**	Russia	0.106	Orange	(E)-2-Decenal	36.29	**●**			**●**	**●**							**●**
**CS07**	Uzbekistan	0.163	Yellow	(E)-2-Decenal	50.53			**●**			**●**	**●**					**●**
**CS08**	Uzbekistan	0.144	Dark yellow	(E)-2-Decenal	48.74		**●**	**●**			**●**		**●**				**●**
**CS09**	Uzbekistan	0.094	Dark yellow	(E)-2-Decenal	42.64	**●**	**●**						**●**		**●**		**●**
**CS10**	Uzbekistan	0.069	Yellow	(E)-2-Decenal	38.68	**●**				**●**				**●**			**●**
**CS11**	Bulgaria	0.157	Orange	(E)-2-Decenal	51.73	**●**	**●**	**●**									**●**
**CS12**	China	0.074	Dark yellow	(E)-2-Decenal	46.74	**●**		**●**			**●**			**●**			**●**
**CS13**	Bhutan	0.307	Milky	(E)-2-Decenal	57.10			**●**			**●**	**●**	**●**				
**CS14**	Costa Rica	0.337	Orange	(E)-2-Decenal	50.62			**●**	**●**	**●**				**●**		**●**	
**CS15**	India	0.100	Red	**Linalool**	30.44		**●**	**●**	**●**	**●**						**●**	
**CS16**	Peru	0.446	Red	(E)-2-Decenal	60.50	**●**		**●**			**●**		**●**			**●**	
**CS17**	Russia	0.156	Dark yellow	(E)-2-Decenal	50.37		**●**	**●**		**●**				**●**		**●**	
**CS18**	Myanmar	0.411	Dark yellow	(E)-2-Decenal	32.38	**●**		**●**					**●**		**●**	**●**	
**CS19**	Mongolia	0.091	Yellow	(E)-2-Decenal	53.48			**●**	**●**	**●**			**●**				
**CS20**	Kosovo	0.091	Yellow	(E)-2-Decenal	51.59			**●**	**●**	**●**							**●**
**CS21**	Kosovo	0.143	Dark yellow	(E)-2-Decenal	52.37						**●**		**●**		**●**		**●**
**CS22**	Kosovo	0.113	Dark yellow	(E)-2-Decenal	44.00		**●**	**●**	**●**	**●**							**●**
**CS23**	Nepal	0.199	Red	(E)-2-Decenal	34.26		**●**	**●**	**●**	**●**							**●**
**CS24**	Kosovo	0.186	Red	(E)-2-Decenal	41.58	**●**				**●**			**●**		**●**	**●**	
**CS25**	Nepal	0.183	Orange	(E)-2-Decenal	33.93	**●**	**●**					**●**				**●**	**●**
**CS26**	Myanmar	0.422	Yellow	(E)-2-Decenal	57.52	**●**					**●**		**●**			**●**	**●**
**CS27**	Ukraine	0.122	Orange	(E)-2-Decenal	53.66	**●**		**●**		**●**	**●**	**●**					
**CS28**	United States	0.083	Yellow	(E)-2-Decenal	44.19	**●**				**●**				**●**			**●**
**CS29**	Kosovo	0.128	Orange	(E)-2-Decenal	46.63	**●**			**●**		**●**						**●**
**CS30**	Kosovo	0.090	Orange	(E)-2-Decenal	58.39			**●**	**●**		**●**					**●**	
**CS31**	Kyrgyzstan	0.276	Orange	(E)-2-Decenal	57.06	**●**		**●**			**●**					**●**	**●**
**CS32**	Uzbekistan	0.083	Red	(E)-2-Decenal	47.95			**●**	**●**								**●**
**CS33**	Uzbekistan	0.075	Orange	(E)-2-Decenal	36.04	**●**	**●**	**●**				**●**					
**CS34**	Uzbekistan	0.070	Yellow	(E)-2-Decenal	45.76	**●**		**●**			**●**		**●**				**●**
**CS35**	Kazakhstan	0.062	Pale yellow	(E)-2-Decenal	40.96	**●**		**●**			**●**	**●**					
**CS36**	Georgia	0.120	Dark yellow	(E)-2-Decenal	30.92	**●**							**●**		**●**	**●**	**●**
**CS37**	Kosovo	0.071	Orange	(E)-2-Decenal	43.59	**●**		**●**				**●**				**●**	**●**
**CS38**	Mongolia	0.143	Orange	(E)-2-Decenal	46.24	**●**					**●**	**●**	**●**				**●**
**CS39**	Mongolia	0.099	Red	**Linalool**	34.52	**●**			**●**	**●**	**●**					**●**	
**CS40**	Kosovo	0.091	Dark yellow	(E)-2-Decenal	46.18		**●**		**●**			**●**		**●**		**●**	
**CS41**	Korea	0.122	Red	**Linalool**	61.12			**●**		**●**	**●**			**●**			
**CS42**	Korea	0.143	Red	(E)-2-Decenal	38.70			**●**	**●**			**●**				**●**	
**CS43**	Korea	0.130	Red	**Linalool**	45.25	**●**		**●**		**●**				**●**		**●**	
**CS44**	Korea	0.178	Dark yellow	(E)-2-Decenal	45.12		**●**	**●**	**●**		**●**					**●**	**●**
**CS45**	Korea	0.156	Yellow	(E)-2-Decenal	50.70	**●**					**●**	**●**	**●**	**●**			**●**
**CS46**	Korea	0.225	Dark yellow	(E)-2-Decenal	44.64	**●**	**●**	**●**		**●**	**●**	**●**					
**CS47**	Korea	0.168	Yellow	(E)-2-Decenal	55.01	**●**						**●**				**●**	
**CS48**	Vietnam	0.169	Orange	(E)-2-Decenal	54.82	**●**		**●**				**●**					**●**

Yield expressed as % (*v*/*w*). Chemotype defined by the compound with the highest relative area (%) in each accession’s essential oil. ●: fragrance type present. Fragrance type abbreviations—Ald: aldehydic; Cit: citrus; Ear: earthy; Flo: floral; Her: herbal; Med: medicinal; Oil: oily; Ori: oriental; Pep: peppery; Spi: spicy; Swe: sweet; Woo: woody. The identification of two Korean accessions (CS41 and CS43) as a linalool chemotype is particularly significant in relation to the PCA results (Section 3.3). These accessions exhibited extremely negative PC1 positions in the score plot, consistent with the strong negative loading of Linalool on PC1 (−0.280). Their elevated Linalool content (45.25–61.12%) substantially increased the within-group dispersion of the Temperate climate zone cluster, demonstrating that chemotypic variation can confound broader biogeographical patterns in multivariate compositional analyses.

**Table 9:** Frequency of fragrance type descriptors assigned to *C. sativum* essential oils by chemotype. Values ≥ 50% in the Linalool chemotype are highlighted.

Fragrance Type	(E)-2-Decenal (*n* = 44) Count	(E)-2-Decenal (%)	Linalool (*n* = 4) Count	Linalool (%)
Earthy	28	64%	**3**	**75%**
Aldehydic	27	61%	**2**	**50%**
Woody	25	57%	0	0%
Medicinal	19	43%	**2**	**50%**
Oriental	15	34%	0	0%
Oily	15	34%	0	0%
Sweet	15	34%	**3**	**75%**
Herbal	14	32%	**4**	**100%**
Floral	13	30%	**2**	**50%**
Citrus	11	25%	1	25%
Peppery	10	23%	**2**	**50%**
Spicy	6	14%	0	0%

Fragrance descriptors were assigned by consensus of three evaluators; a descriptor was recorded as present when agreed upon by at least two of three evaluators. Multiple fragrance types could be assigned per accession. *n* = 44 ((E)-2-Decenal chemotype); *n* = 4 (Linalool chemotype).

**Table 10:** Country of origin and Köppen–Geiger climate zone of 48 *C. sativum* accessions.

Origin	Köppen–Geiger Zone	Samples
Nepal	Tropical/Subtropical	CS01, CS02, CS23, CS25
Kosovo	Temperate	CS03, CS20, CS21, CS22, CS24, CS29, CS30, CS37, CS40
Korea	Temperate	CS04, CS05, CS41, CS42, CS43, CS44, CS45, CS46, CS47
Russia	Continental/Cold	CS06, CS17
Uzbekistan	Arid/Semi-arid	CS07, CS08, CS09, CS10, CS32, CS33, CS34
Bulgaria	Temperate	CS11
China	Arid/Semi-arid	CS12
Bhutan	Tropical/Subtropical	CS13
Costa Rica	Tropical/Subtropical	CS14
India	Tropical/Subtropical	CS15
Peru	Tropical/Subtropical	CS16
Myanmar	Tropical/Subtropical	CS18, CS26
Mongolia	Arid/Semi-arid	CS19, CS38, CS39
Ukraine	Temperate	CS27
United States	Temperate	CS28
Kyrgyzstan	Arid/Semi-arid	CS31
Kazakhstan	Arid/Semi-arid	CS35
Georgia	Temperate	CS36
Vietnam	Tropical/Subtropical	CS48

## Data Availability

The data presented in this study are available on request from the corresponding author.

## Supplementary Materials

The following supporting information can be downloaded at: https://www.mdpi.com/article/10.3390/molecules31111950/s1.
